# Programmable optical encryption using thickness-controlled stretchable chiral liquid crystal elastomers

**DOI:** 10.1038/s41377-025-01815-z

**Published:** 2025-03-26

**Authors:** Seungmin Nam, Seohyun Woo, Ji Yoon Park, Su Seok Choi

**Affiliations:** 1 Department of Electrical Engineering, Pohang University of Science and Technology (POSTECH), 77 Cheongam-ro, Pohang, 37673 Korea; 2 Department of Electrical Engineering, Chosun University, 17, Chosundae 1-gil, Gwangju, 61452 Korea

**Keywords:** Liquid crystals, Optical sensors, Optical data storage

## Abstract

The growing demand for cryptographic security encourages the innovation of advanced materials with unique optical properties to secure information using light. Structural colors with soft materials exhibit dynamically tunable optical properties in response to external stimuli, making them ideal for multi-level photonic encryption. However, most previous studies on structural color-based photonic encryption have predominantly focused on single-wavelength tuning while employing inadequate triggering methods for practical device applications. Here, we propose a chiral liquid crystal elastomer (CLCE) designed for stretching-induced multi-wavelength control to enhance photonic encryption functionality. By employing a heterogeneous configuration with thickness-modulated CLCE, we achieve multi-photonic band wavelength control under mechanical deformation. Furthermore, this method extends the tunable wavelength range beyond the visible spectrum into the infrared region and integrates a discrete multi-pixel array structure, enabling advanced spatial and spectral control for complex encryption schemes. This multi-wavelength modulation method is expected to provide significant potential for applications in photonic encryption, adaptive optics, and next-generation information security systems.

## Abstract (web summary)

By utilizing chiral liquid crystal elastomers with different thicknesses, multiple photonic band control was achieved by mechanical deformation, enabling the proposal of multi-level programmable optical encryption.

## Graphical abstract

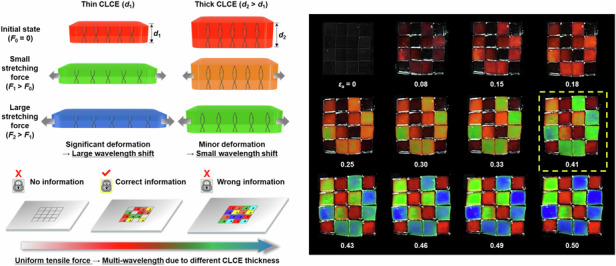

## Introduction

In the era of digital information, ensuring the security of data transmission and storage has become a critical priority^1–4^. As technology advances, conventional cryptographic methods are increasingly vulnerable to the growing computational power and the impending development of quantum computing^5,6^. This susceptibility necessitates the exploration of novel encryption techniques that utilize advanced materials with unique physical properties. Among these innovative approaches, photonic encryption—which uses light to encode information—offers a promising solution in this regard^7–9^. Notably, structural colors in soft materials, which exhibit dynamically tunable optical properties in response to external stimuli, are emerging as a viable option for advanced multi-level photonic encryption^10,11^.

Structural colors are generated through the interaction of light with micro- or nanostructures within periodic dielectric material, rather than light absorption from pigmentation^12–14^. This light interaction can be modulated by changing the periodic structure of the material, leading to alterations in the wavelength of structural color^15–17^. Among various structural-colored materials, chiral liquid crystal elastomers (CLCEs) are particularly gaining attention^18–21^ as attractive materials due to their flexibility, scalability, and responsiveness to external stimuli, such as temperature^22–24^, electric fields^25–28^, and mechanical deformation^29–32^. These properties allow CLCEs to undergo significant changes in their optical characteristics, making them ideal candidates for dynamic photonic encryption applications.

Due to the advantages of CLCEs as structural-colored-materials, many studies have explored their potential for applications in structural color-based photonic encryption technologies. However, there are still two major technical limitations in CLCE-based photonic encryption. The first of these is the lack of practical triggering methods. Most research on photonic encryption using CLCE structural color involves encoding and tuning data through responses to temperature, humidity, or chemical solvents^33,34^. However, this approach requires additional components for the user to apply external stimuli to the encryption device, limiting the ability to achieve faster and more versatile tuning. If the user could directly stretch the encryption device and encode various information based on the amount or mode of stretching, it would eliminate the need for the aforementioned additional elements and allow for faster data transitions, offering significant advantages.

The second limitation is that most multi-level photonic encryption studies using structural-colored materials have focused primarily on simple single-wavelength tuning and single-pixel configuration^35–37^. In contrast, multi-wavelength control, where multiple colors can be tuned independently, represents a major advancement in this field. By expanding the range of tunable colors and enabling independent control of each unit, multi-wavelength control allows for more complex and secure encryption schemes.

The technology of multi-wavelength separation using stretchable structural colors, including CLCEs, can be broadly classified into two major approaches. One approach involves controlling the local strain by adjusting the thickness of the backing layer supporting the structural color material, as reported by Miller et al.^38^. However, this method is challenging to apply in practical applications due to the reduced flexibility of the entire device, as well as increased overall weight and thickness. The other approach involves controlling the elastic modulus by adjusting the crosslink density of the structural color material itself, thereby separating the color shift occurring under the same mechanical stress. This method was researched by our group^39^ and by de Castro et al.^40^. Despite its potential, this method also has drawbacks, such as the complexity of the synthetic manufacturing process required to design structural color materials with different crosslink densities and the limited amount of color separation achieved.

Moreover, conventional studies on CLCE structural colors typically rely on single-pixel or single-pattern designs, where a fixed mask determines a predetermined pattern. As a result, they are limited to simple information representation, where only color changes occur within pre-defined patterns. However, to expand the number of possible information combinations and achieve greater integration of encoded information, it is essential to move beyond a single film, and single-wavelength change in conventional approaches^34–37,41–44^ and adopt a discrete pixel structure that enables independent control of individual CLCE pixels and precise wavelength separation.

In response to this challenge, we propose a novel approach to achieve multi-level photonic encryption devices using stretching-induced multi-wavelength modulation utilizing heterogeneous design of CLCEs. By designing a device configuration with modulation of the CLCE thickness, we can independently control multiple wavelengths under the uniform tensile force. Specifically, when the same tensile force is applied to CLCEs designed with the same elastic modulus and initial central wavelength but different thicknesses, the thicker CLCE experiences lower tensile stress, resulting in a smaller wavelength shift. Conversely, when the same tensile force is applied to a thinner CLCE, it undergoes higher tensile stress, leading to a larger wavelength shift. Furthermore, our method utilizes discrete pixel structures to significantly expand the potential for information representation. By employing individually tunable discrete pixels arranged in a modular array, we enable the creation of more complex patterns and multi-level information. This innovation moves beyond the static, pre-defined patterns seen in traditional approaches, offering a system that can dynamically adapt to different encryption scenarios and applications

In other words, unlike previous studies that primarily focused on single-wavelength tuning of photonic band gaps and single-pixel configurations^37,44^, this work introduces a novel approach for multi-wavelength modulation by leveraging the thickness-dependent mechanochromic response in a pixel array configuration of CLCEs. This method not only enables simultaneous control over multiple wavelengths under a uniform tensile force but also extends the tunable wavelength range beyond the visible spectrum into the infrared region. By doing so, it significantly enhances the complexity and security of photonic encryption applications, offering greater versatility and broader functionality for advanced encryption technologies. The potential applications of this mechanical stretching-based multi-wavelength control technology extend far beyond photonic encryption. This technology is also highly promising for adaptive optics, sensors, displays, and smart materials, due to its capability to finely and independently tune optical properties in response to mechanical forces. The innovative use of heterogeneous CLCEs for multi-wavelength control overcomes the limitations of single-color tuning, enabling more secure and complex encryption schemes.

## Results

**Fig. 1:**
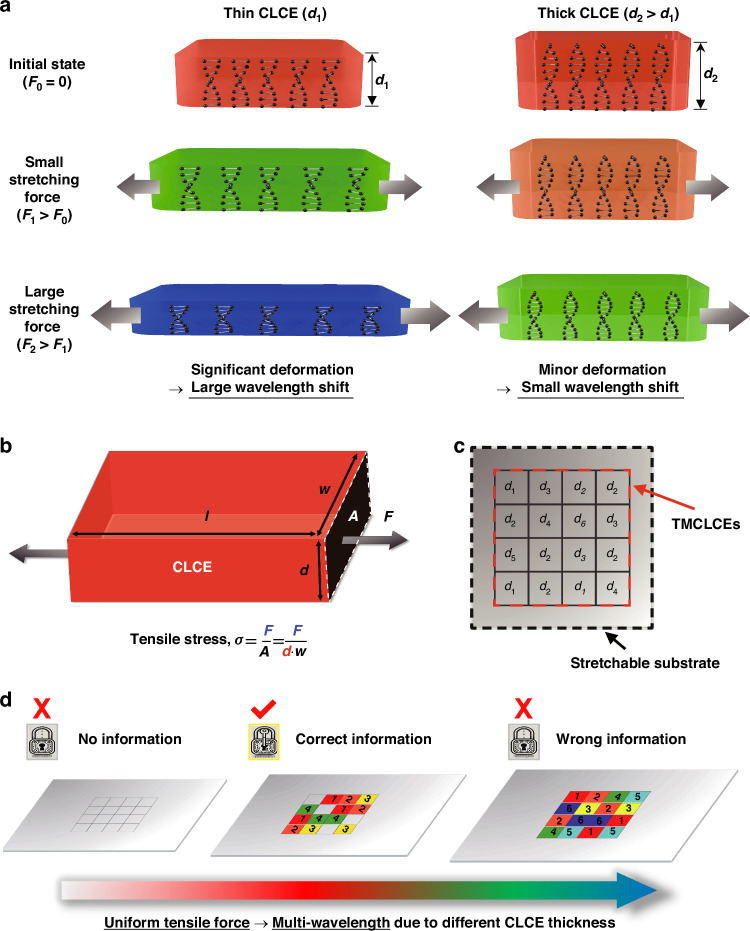
Schematic of the multi-wavelength control and stretchable photonic encryption device using thickness-modulated chiral liquid crystal elastomers (TMCLCEs) **a** CLCEs with different thicknesses (*d*_1_ < *d*_2_) exhibit varying wavelength shifts under tensile force, with thinner CLCEs experiencing greater stress and larger shifts. **b** The amount of tensile stress in a TMCLCE samples tensile force. **c** TMCLCEs are arranged in a pixel array for photonic encryption device. **d** The encrypted information is displayed based on the applied strain: no strain (locked), appropriate strain (unlocked), and excessive strain (locked)

The operational principle of the multi-wavelength control and stretchable photonic encryption applications using thickness-modulated chiral liquid crystal elastomers (TMCLCEs) is illustrated in Fig. 1. To achieve multi-wavelength separation, one can consider fabricating CLCEs with the same initial color and elastic modulus but different configuration in CLCE thicknesses (thin CLCE, thick CLCE) with different thicknesses (*d*_1_ < *d*_2_) (Fig. 1a). In their unstrained state, both CLCEs exhibit similar optical properties. Upon the application of a uniform tensile force, the thinner CLCE experiences higher tensile stress, resulting in a more significant wavelength shift compared to the thicker CLCE. This differential mechanochromnic response allows for complicated control of multiple wavelengths under the same tensile force, enhancing the complexity of the encoded information.

Specifically, when the same force is applied to TMCLCEs, the difference in cross-sectional area caused by different thickness can result in varying amounts of tensile stress (Fig. 1b). The tensile stress (*σ*) is inversely proportional to the thickness (*d*) of the CLCE, as described by the equation as follows:

1 $$\sigma =\frac{F}{A}=\frac{F}{d\cdot w}$$

Where *A* is the cross-sectional area, *F* is the tensile force, and *w* is the width of the CLCE. CLCEs fabricated with different thicknesses (*d*) will have larger cross-sectional areas under identical CLCE geometric conditions (*l*, *w*, *d*). Therefore, when the same tensile force (*F*) is applied, a smaller amount of tensile stress (*σ*) will be generated in thicker CLCE. The relationship between tensile strain and tensile stress is given by:

2 $$\varepsilon =\frac{\sigma }{E}$$

where *ε* is the tensile strain, and *E* is the elastic modulus. Assuming the elastic modulus is also designed to be the same in all TMCLCEs, it can be intuitively understood that different tensile stresses will result in different CLCE tensile strains. In structural-colored materials, tensile strain corresponds to the shift amount of the central wavelength. Therefore, by fabricating CLCEs with the same conditions except for the thickness, different color shifts can be achieved under the same tensile force. This hypothesis demonstrates the feasibility of using TMCLCEs to achieve multi-wavelength control. By precisely designing the thickness of the CLCEs, we can independently control the wavelength shift of each coloration unit under a uniform tensile force (Supplementary Note 1). This capability allows for the encoding of information at multiple levels, significantly increasing the complexity and security of photonic encryption.

To create a multi-level encryption device using the multi-wavelength control mechanism of CLCEs, we arranged transparent TMCLCEs, designed with an initial central wavelength in the IR region, on a stretchable substrate in a pixel array configuration (Fig. 1c). Here, *d*_n_ (*n* = 1,2, ⋯) represents the thickness of each different CLCE. Utilizing this configuration, no information is displayed in the initial state, but when stretching deformation is applied, a device capable of displaying multiple levels of information depending on the tensile strain is realized (Fig. 1d). Specifically, when the user stretches the substrate embedded with TMCLCEs, the thinner CLCEs quickly shift from the IR region to the visible region and display information, while the thicker CLCEs remain transparent until significant deformation is applied. In this context, the initial state is defined as having no information (locked/no information), the state with appropriate deformation applied as unlocked (correct information), and the state with excessive deformation as locked again (wrong information).

**Fig. 2:**
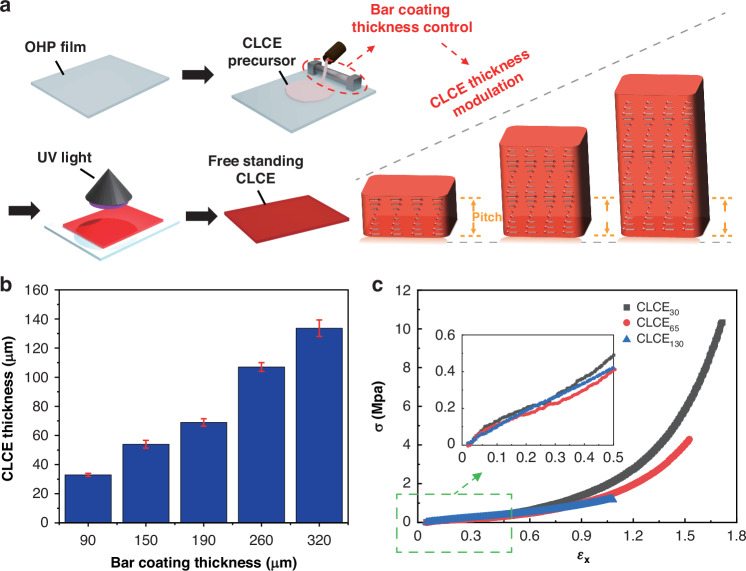
**Fabrication and mechanical analysis of thickness-modulated chiral liquid crystal elastomers (TMCLCEs)** **a** Schematic of the fabrication process for TMCLCEs, utilizing bar coating thickness control for precise modulation of CLCE thickness. **b** Measured the relationship between bar coating thickness and resulting CLCE thickness, demonstrating systematic control over geometric dimensions. **c** Stress-strain curves for TMCLCEs with different thicknesses, confirming similar elastic moduli across samples. Inset: Initial elastic region further illustrating the consistent mechanical behavior across thickness variations

To achieve multi-wavelength control, we fabricated CLCEs with identical initial structural colors and elastic moduli but with varying thicknesses (Fig. 2). The fabrication process involves spreading the CLCE precursor on a substrate followed by modulating the thickness of the bar coating process (Fig. 2a). This method allows precise control over the thickness of the CLCEs while maintaining the same initial optical and mechanical properties. The synthesis of CLCE precursor was carried out using the well-known thiol-acrylate reaction method^45,46^ (Table S1, Fig. S3).

The thicknesses of TMCLCEs are measured to investigate the relationship between bar coating thickness and the resulting CLCE thickness (Fig. 2b). As expected, there is a clear correlation between the coating thickness and the fabricated CLCE thickness, enabling systematic control over the geometric dimensions of material by adjusting bar coating thickness. This precise thickness modulation is crucial for achieving the desired multi-wavelength modulation under mechanical stretching. When applying tensile force to TMCLCEs, to generate tensile strain inversely proportional to thickness, the elastic modulus ideally must be the same for desired multi-wavelength control (see Eqs. (1), (2) and Supplementary Note 1). If the elastic modulus varies with thickness, it would be difficult to definitively claim that multi-wavelength separation occurs purely due to thickness changes, making precise wavelength control unattainable. In this regard, we measured the stress-strain curves of CLCEs with different thicknesses and confirmed that they have almost identical elastic modulus values regardless of thickness (Fig. 2c, Fig. S4). The inset of Fig. 2c further illustrates the initial elastic region, showing almost uniform stress-strain relationships for different thicknesses. Since the elastic modulus of CLCE remains consistent across different thicknesses, we confirm that precise wavelength controllability can be achieved by adjusting the thickness of the CLCE.

**Fig. 3:**
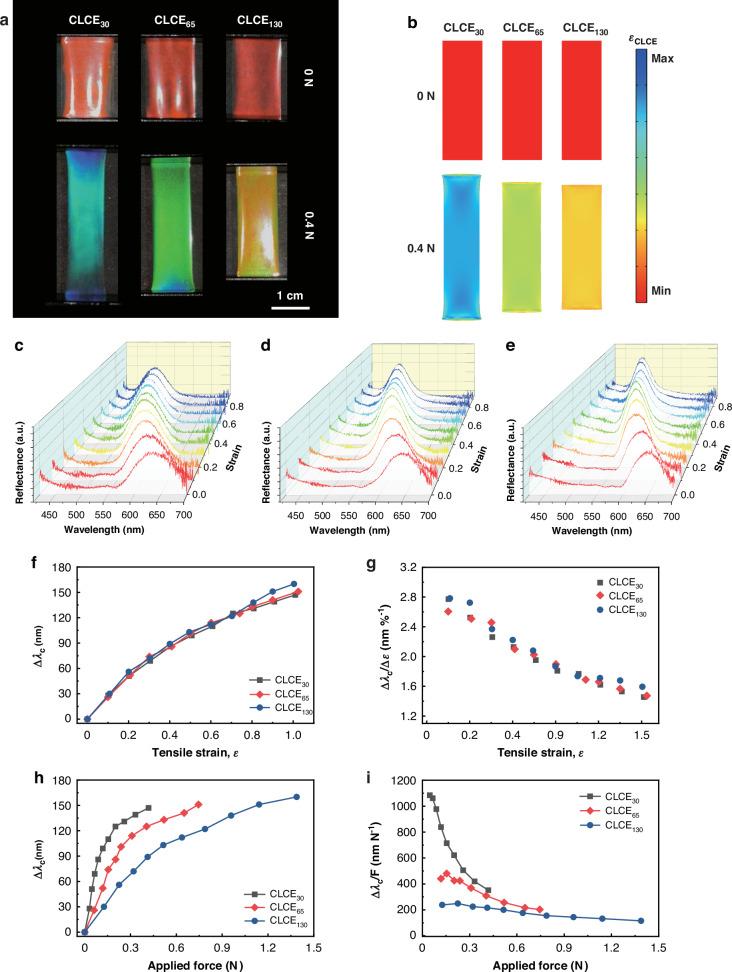
**Mechanochromic response of free-standing thickness-modulated chiral liquid crystal elastomers (TMCLCEs) under uniaxial stretching** **a** Photographs of free-standing TMCLCEs with thicknesses of 30 µm (CLCE_30_), 65 µm (CLCE_65_), and 130 µm (CLCE_130_) under no load (0 N) and a tensile force of 0.4 N. **b** Finite element analysis (FEA) showing the strain distribution in TMCLCEs under the same tensile force of 0.4 N. **c**–**e** Reflectance spectra of CLCE_30_ (**c**) CLCE_65_ (**d**) and CLCE_130_ (**e**) under increasing tensile strain, showing a shift in the reflected wavelength as the strain increases. **f** Wavelength shift versus tensile strain for the different TMCLCEs. **g** Wavelength shift per unit tensile strain (Δ*λ*_c_/*ε*) against the applied strain. **h** Wavelength shift versus tensile stress for the different CLCEs, illustrating the significant dependence of wavelength tuning on CLCE thickness. **i** Wavelength shift per unit tensile force (Δ*λ*_c_/*F*) against the applied force

To investigate the feasibility of multi-wavelength control in TMCLCEs, we fabricated samples with the same initial red colors and elastic moduli but with varying thicknesses of 30 µm (CLCE_30_), 65 µm (CLCE_65_), and 130 µm (CLCE_130_). After synthesizing CLCEs with different thicknesses, applying the same tensile force allows us to investigate the influence of mechanical deformation on the mechanochromic property separation of free-standing TMCLCEs (Fig. 3). When a tensile force of 0.4 N is applied to each CLCE, the TMCLCE samples exhibit distinct multi-wavelength shifts in structural color depending on their thickness (Fig. 3a). Specifically when a tensile force of 0.4 N is applied, the CLCE_30_ sample shifts from its initial red to blue-green color, while the thicker CLCE_130_ sample only shifts from the same initial red to orange-yellow color. This observation highlights the role of thickness in realizing the multi-wavelength modulation of the TMCLCEs under uniform mechanical deformation. The strain distribution for the TMCLCE samples under no applied force (0 N) and a tensile force of 0.4 N are calculated using finite element analysis (FEA) (Fig. 3b, Video S1). These simulations reveal that thinner CLCEs experience higher strain levels compared to thicker ones. This increased strain in thinner samples correlates with the larger wavelength shifts observed experimentally, confirming that thickness modulation effectively controls the optical response of the CLCEs. Reflectance spectra for the CLCE samples with different thicknesses under varying strain levels (Fig. 3c–e) provide further insight into the wavelength shifts. As the strain increases, the reflectance peaks consistently shift towards shorter wavelengths for all samples, indicating a blue shift. It should be noted that the same tensile force applied to TMCLCEs induces a multi-wavelength shift. However, the observation that the mechanochromic properties are consistent with applied tensile strain suggests that the structural color change in CLCEs is proportional to the magnitude of the applied deformation (see Supplementary Note 1). Based on measured reflectance spectra for the CLCE samples, the central wavelength shift in response to the applied tensile strain was observed (Fig. 3f). The wavelength change did not show any significant variation in trend with respect to the thickness of the CLCE under the applied tensile strain. This is because the central wavelength shift of the CLCE is ultimately determined by the change in pitch length, which is affected not by the absolute thickness of the CLCE but by the thickness change ratio. Therefore, the strain-color sensitivity (Δ*λ*_c_/*ε*) exhibits a similar trend across the entire strain range, regardless of the CLCE thickness (Fig. 3g). We also observe the relationship between the applied force and the wavelength shift for CLCE samples with different thicknesses (Fig. 3h). It is evident that as the applied force increases, the wavelength shift amount also increases for all samples. Notably, the 30 µm thick CLCE_30_ exhibits the largest wavelength shift, followed by the CLCE_65_ and CLCE_130_ samples under identical amounts of applied tensile force. This indicates that thinner CLCEs are more responsive to the applied force, showing a greater optical tuning range. As expected, since greater strain and the resulting wavelength shift occur under the same tensile force, thinner samples exhibit higher force-color sensitivity (Δ*λ*_c_/*F*) across all force ranges (Fig. 3i).

**Fig. 4:**
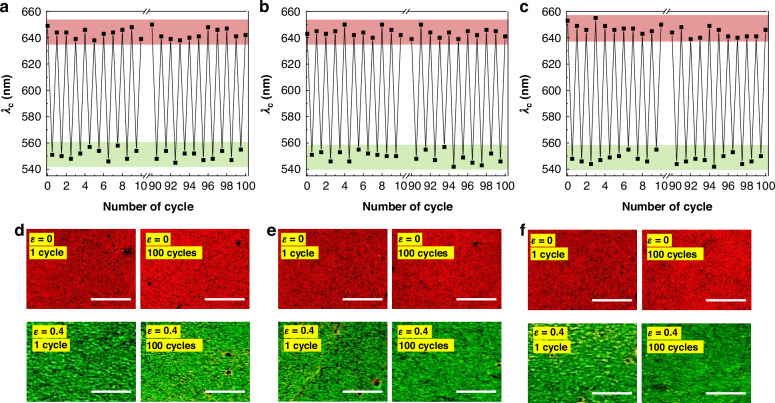
**Durability and repeatability of chiral liquid crystal elastomers (CLCEs) with varying thickness under cyclic mechanical deformation** **a**–**c** Optical wavelength change of CLCEs with thickness of 30 µm (CLCE_30_) 30 µm (**a**) 65 µm (CLCE_65_) (**b**) and 130 µm (CLCE_130_) (**c**) during 100 cycles of tensile strain (*ε* = 0 and *ε* = 0.4). **d**–**f** Microscopic images of CLCEs with thicknesses of 30 µm (CLCE_30_) (**d**) 65 µm (CLCE_65_) (**e**) and 130 µm (CLCE_130_) (**f**) under no strain (*ε* = 0) and a tensile strain of *ε* = 0.4, both before after 1 cycle and 100 cycles stretching, demonstrating the physical and optical stability of the material (Scale bar: 50 µm)

To verify whether TMCLCEs possess the necessary reliability for device applications, the durability and repeatability of their optical properties under cyclic mechanical deformation were systematically investigated (Fig. 4). The central wavelength changes under no strain (*ε* = 0) and a tensile strain of *ε* = 0.4 was observed during 100 cycles of stretching for CLCEs with varying thicknesses of 30 µm, 65 µm, and 130 µm, respectively (Fig. 4a–c). The cycle tests indicate that all CLCE samples maintain their structural color properties effectively, showing minimal degradation or shift in wavelength after repeated cycles of strain. This consistency is essential for maintaining the integrity of encrypted information in photonic encryption systems and for the precise tuning of optical properties in adaptive optics and sensor technologies.

Polarized optical microscopy images in reflection mode of the CLCEs with different thicknesses of 30 µm, 65 µm, and 130 µm taken under no strain (*ε* = 0) and a tensile strain of *ε* = 0.4 after 1 and 100 cycles are also observed to provide visual confirmation of the material stability in microscale (Fig. 4d–f). The images show that the physical structure and color of the CLCEs remain largely unchanged after repeated 100 cycles of stretching. There is no significant morphological damage or color degradation, indicating that the materials can withstand mechanical stress without compromising their functional properties. This robustness is attributed to the intrinsic properties of the CLCEs and the precise control over their initial optical characteristic and crosslinking density during fabrication.

The mechanical and optical properties of TMCLCEs with varying bar coating thicknesses were systematically analyzed, as summarized in Table 1. The table provides a comprehensive summary of the measurements conducted on CLCE samples with varying thicknesses. The bar coating thickness, sample thickness (*d*), elastic modulus (*E*), strain-color sensitivity (Δ*λ*_c_/*ε*), force-color sensitivity (Δ*λ*_c_/*F*), and tuning repeatability are all detailed for three different TMCLCE samples: CLCE_30_, CLCE_65_, and CLCE_130_. The sample thicknesses were controlled through the bar coating process, resulting in mean values of 30.1 ± 1.0 µm, 64.7 ± 2.5 µm, and 129.6 ± 5.7 µm for CLCE_30_, CLCE_65_, and CLCE_130_, respectively. The elastic modulus of the samples, which is a critical factor in determining their mechanical response, showed slight variations across the different thicknesses, with values ranging from 0.82 MPa to 0.90 MPa. The strain-color sensitivity (Δ*λ*_c_/*ε*) for the samples varied between 1.5 and 2.8 nm%^−1^, indicating a consistent response to tensile strain across different thicknesses. Notably, the force-color sensitivity (Δ*λ*_c_/*F*) showed a more significant variation, with thinner samples (CLCE_30_) exhibiting higher sensitivity (351.7 ~ 1085.1 nm N^−1^) compared to thicker samples (CLCE_130_), which showed lower sensitivity (115.3 ~ 248.9 nm N^−1^). This indicates that thinner samples are more responsive to applied forces, resulting in greater wavelength shifts. All samples demonstrated excellent tuning repeatability, maintaining their optical properties for up to 100 cycles.

**Table 1:** Mechanical and optical properties of thickness-modulated chiral liquid crystal elastomers TMCLCEs

Sample	Bar coating thickness	Sample thickness (*d*)	Elastic modulus (*E*)	Strain-color sensitivity (Δ*λ*_*c*_/*ε*)	Force-color sensitivity (Δ*λ*_*c*_/*F*)	Tuning repeatability
CLCE_30_	90 μm	30.1 ± 1.0 μm	0.84 MPa	1.5 ~ 2.8 nm %^−1^	351.7 ~ 1085.1 nm N^−^^1^	Repeatable (up to 100 cycles)
CLCE_65_	190 μm	64.7 ± 2.5 μm	0.90 MPa	1.6 ~ 2.8 nm %^−1^	202.7 ~ 480.5 nm N^−1^	Repeatable (up to 100 cycles)
CLCE_130_	320 μm	129.6 ± 5.7 μm	0.82 MPa	1.5 ~ 2.6 nm %^−1^	115.3 ~ 248.9 nm N^−1^	Repeatable (up to 100 cycles)

Summary of bar coating thickness, sample thickness (*d*), elastic modulus (*E*), strain-color sensitivity (Δ*λ*_c_/*ε*), force-color sensitivity (Δ*λ*_c_/*F*), and tuning repeatability for CLCEs of different thicknesses (30 µm, 65 µm, 130 µm) under an applied tensile strain of *ε* = 0.4

**Fig. 5:**
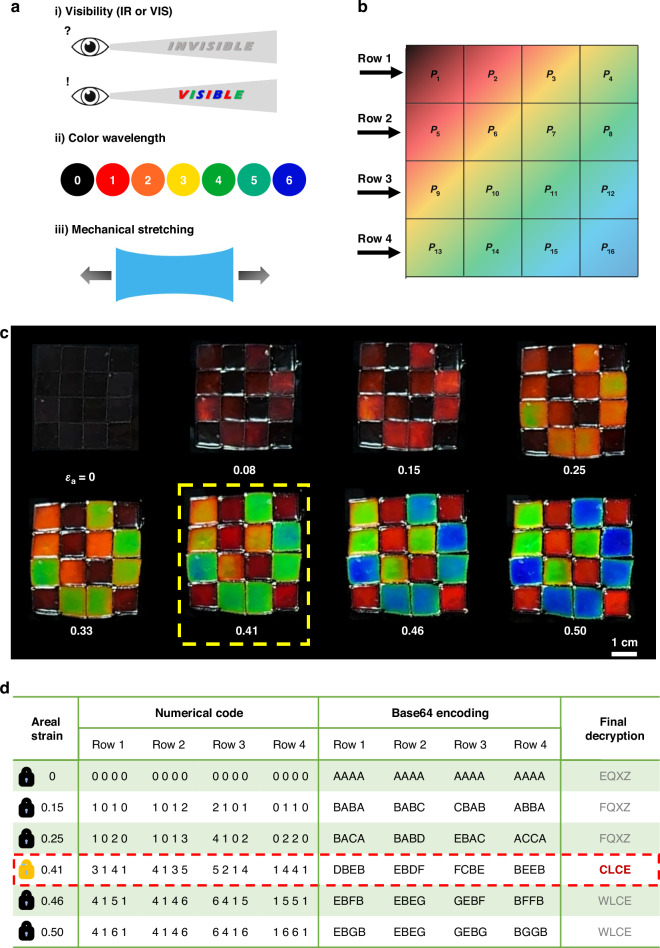
**Stretchable multi-level photonic encryption system using thickness-modulated chiral liquid crystal elastomer (TMCLCE) pixels** **a** Multi-level features of proposed photonic encryption device. **b** 4 × 4 pixel configuration using TMCLCEs for multi-level photonic encryption using mechanical stretching. **c** Color shift of the TMCLCE pixels under increasing biaxial strain (*ε*_a_). At *ε*_a_ = 0.41, the correct encryption pattern emerges, while at other strain values (0, 0.08, 0.15, 0.25, 0.33, 0.46, and 0.50), incorrect or incomplete information is displayed. **d** The pixel pattern is decoded into numerical codes using Base64 and a lab-made substitution table, with *ε*_a_ = 0.41 yielding the correct string “CLCE.” Other strain levels result in meaningless strings, demonstrating the selective information revealed through mechanical deformation

Using the TMCLCEs enabled for multi-wavelength modulation, we proposed a stretchable multi-level photonic encryption application (Fig. 5). Embedding CLCEs with varying thicknesses, designed with a central wavelength in the IR region (Fig. S5), into a transparent stretchable substrate forms a single device where multi-wavelength control is achieved through mechanical stretching (Table S1, Fig. S6). When mechanical stretching is applied to the substrate of this multi-level encryption device, thicker CLCE pixels require more substrate strain to shift the wavelength into the visible region, whereas thinner CLCE pixels shift to the shorter wavelengths of the visible region much faster.

The information in the TMCLCE-based multi-level encryption device can be encoded through three distinct functionalities (Fig. 5a). Firstly, by designing the initial central wavelength of the CLCE in the near-infrared region, no pattern is visible at the initial unstretched state (visibility). Secondly, the photonic encryption pattern allows for various decoding options based on color information (color wavelength). Thirdly, a large amount of diverse information can be stored depending on the stretching strain and stretching mode (mechanical stretching).

By using the multi-wavelength control method by adjusting the thickness of the CLCE and incorporating the three functionalities described above, a stretchable multi-level photonic encryption device can be fabricated. TMCLCEs with an initial wavelength designed in the IR region were embedded in a stretchable substrate in a pixel binning configuration, creating 4 × 4 TMCLCE pixels (Fig. 5b). When biaxial areal strain is applied to the TMCLCE pixels embedded in the stretchable substrate, a multi-level encryption system can be achieved, where each pixel separates into multi-wavelengths (Fig. 5c, Video S2). In the initial state without strain applied to substrate (*ε*_a_ = 0), all CLCE pixels have their central wavelengths in the IR region, resulting in a “no information” state. As biaxial strain is applied, the central wavelengths of the CLCE structural colors begin to shift to visible wavelength bands with respect to CLCE thickness. Specifically, when the areal strain applied to the substrate reaches 0.15 (*ε*_a_ = 0.15), some CLCEs tune to the red and orange regions, displaying partial information, while the thicker CLCEs still maintain their central wavelengths in the IR region. This state, while displaying some information, does not show the correct information and is referred to as the “wrong information state.” At *ε*_a_ = 0.41, a well-defined multicolored pattern emerges, corresponding to the desired encrypted information. This strain state, where the TMCLCE pixels represent the correct desired pattern, is referred to as the “correct information state.” The information displayed at this specific areal strain value is the only valid information encoded in the device. As the areal strain value exceeds 0.41, reaching 0.46 or 0.50, a vivid multi-wavelength pattern reappears due to the thickness differences of TMCLCEs, but since it does not represent the correct information, it is defined as the “wrong information state.”

The TMCLCE pixel data can be decoded into numerical code based on the following two rules. First, each pixel is assigned a numerical value corresponding to its color: transparent-IR (0), red (1), orange (2), yellow (3), green (4), sky blue (5), and blue (6) (Fig. 5a). The data is then read row by row and represented as a numerical code in a 4 by 4 matrix format (Fig. 5b). The numerical code obtained through this process is then converted into a string using Base64 encoding (Fig. S7). This string can be further decoded through a second step by applying a Lab-made string substitution table (Fig. S7).

The pixel data for each strain level is quantified in Fig. 5c, where the 16 pixels (*P*_1_ to *P*_16_) are organized into a row-and-column matrix and decoded based on their corresponding colors. For instance, at *ε*_a_ = 0.41, the pixel array reveals unique numerical codes in which the colors are decoded as follows: (Row 1; Row 2; Row 3; Row 4) = (3141; 3125; 5214; 0441). This numerical code is converted into the following string using the well-known Base64 index table: (Row 1; Row 2; Row 3; Row 4) = (DBEB; DBCF; FCBE; AEEB). Then, applying the inhouse Lab-made substitution table (CSS decryption table), the final information represented by the CLCE pixel configuration at *ε*_a_ = 0.41 is decoded as “CLCE.” Please refer to Fig. S3 in the Supplementary Information for each of the substitution tables. In contrast, the final decoded information at other strain values results in meaningless strings as follows: (*ε*_a_, decoded string) = (0, EQXZ), (0.15, FQXZ), (0.25, FQXZ), (0.46, WLCE), (0.50, WLCE).

This process demonstrates that the TMCLCE-based encryption system can effectively conceal information in its initial IR-reflective state, revealing the correct encrypted data only under specific mechanical deformation. The gradual color shift with applied strain enables the encoding of multiple layers of information, while the selective display of the correct data at *ε*_a_ = 0.41 enhances the system security and precision. The color-to-string mapping further validates this approach by translating the visible pattern into numerical data, followed by string information that forms the encryption key. The ability to encode and decode information through mechanical strain and color-based interpretation provides a robust framework for advanced encryption technologies. This mechanically tunable photonic system offers great potential for secure data storage and retrieval, ensuring that only the correct strain can unlock the intended information.

## Discussion

With rising security demands, conventional encryption faces vulnerabilities from advancing computational and quantum technologies, sparking interest in photonic encryption, which encodes data via light. CLCEs, with tunable optical properties responsive to temperature, electric fields, and mechanical strain, hold strong potential for multi-level encryption applications. However, current CLCE-based photonic encryption systems have limitations: they typically use only single-wavelength tuning and need external triggering components. Enabling multi-wavelength modulation direct mechanical control, and advanced multi-pixel configurations in CLCE-based photonic encryption could support faster, more secure, and more complex encryption schemes.

In this study, we demonstrated a novel multi-wavelength control technique using TMCLCEs for advanced photonic encryption. By embedding CLCEs with varying thicknesses into a stretchable substrate, we achieved precise wavelength separation and control through mechanical stretching. The thickness variation resulted in distinct color shifts under the same tensile force, with thinner CLCEs exhibiting larger wavelength shifts than thicker ones. This allowed us to encode multiple layers of information in a single device, significantly enhancing the complexity and security of the encrypted data. Furthermore, the use of a discrete multi-pixel array structure enabled advanced spatial and spectral control, allowing for the creation of complex patterns and significantly expanding the encoding capacity. This modular design moves beyond single-pixel or static pattern approaches, enabling independent control of each pixel and dynamic adaptation to diverse encryption scenarios.

Additionally, unlike conventional systems requiring external triggering components, our approach allows direct mechanical stretching to adjust color states, simplifying device architecture and reducing potential failure points. Notably, our method extends the tunable wavelength range beyond the visible spectrum into the infrared region, offering greater versatility and functionality for advanced encryption technologies. This direct tunability enables immediate, more adaptive data transitions, dynamically revealing or concealing information based on applied strain.

In summary, this study establishes the viability of TMCLCEs in multi-level photonic encryption, offering a versatile platform for applications where secure, dynamic, and visually encoded information is essential. By integrating thickness-based modulation, multi-pixel configurations, and extended wavelength control, our framework significantly advances the functionality and security of photonic encryption systems. The demonstrated technology holds promise for next-generation encryption devices, with broad implications for advancing information security standards in an increasingly digital era.

## Materials and methods

### Materials

The chiral reactive mesogen (3 R,3aS,6aS)-hexahydrofuro[3,2b]furan-3,6-diyl bis(4-(4-((4-(acryloyloxy)butoxy)carbonyloxy)benzoyloxy)benzoate) (LC756) was obtained from BASF (Ludwigshafen, Germany). The liquid crystalline reactive mesogen 1,4-bis-[4-(3-acryloyloxypropyloxy)benzoyloxy]-2-methylbenzene (RM257) was sourced from GrandinChem (Qingdao, China). The thiol monomer, 2,2′-(ethylenedioxy)diethanethiol (EDDET), along with the thiol crosslinker pentaerythritol tetrakis(3-mercaptopropionate) (PETMP), the photoinitiator 2,2-dimethoxy-2-phenylacetophenone (Irgacure 651), the catalyst dipropylamine (DPA), and anhydrous toluene (99.8%) were supplied by Sigma Aldrich (St. Louis, Missouri, USA). The OHP film was provided by Copierland (Paju, South Korea), and the VHB 4910 adhesive film, used for the stretchable substrate, was obtained from 3 M (Maplewood, Minnesota, USA).

### Fabrication of TMCLCEs

LC756 and RM257 were first dissolved in toluene and heated to 80 °C for 10 minutes to ensure a uniform mixture. After the solution was allowed to cool to room temperature, EDDET, PETMP, Irgacure 651, and DPA were added, and the mixture was thoroughly combined using a vortex until it reached the proper viscosity for bar coating (quantities specified in Table S1). This blending process typically takes about 10 minutes. The solution was then applied to the OHP film with a gap applicator at a speed of 10.7 mm/s. As soon as the intended reflective color emerged, the film was subjected to photo-polymerization under UV light (30 mW/cm^2^, 365 nm) for 2 minutes in a UV chamber (CSM1010, AUVCURE). This procedure was repeated to produce TMCLCEs, each with varying bar coating thicknesses.

### Mechanical and optical characterization of TMCLCEs

To perform mechanical characterization of TMCLCEs, tensile stress was precisely applied using the MFS350 modular force stage (Linkam Scientific Instruments, Salford, UK). For the mechanochromic analysis of both TMCLCEs and the developed multi-level encryption devices, optical measurements were conducted using a microscope setup that included the BX53M (Olympus, Shinjuku, Japan), a HAWK-SCM63 CMOS camera (Zootos, Darien, CT, USA), and an HR4 Pro high-resolution spectrometer (Ocean Insight, Orlando, FL, USA), while the stretching deformation was applied through the modular force stage.

### Finite element analysis

We verified the multi-wavelength separation in TMCLCEs due to adjustments in the thickness using FEA, conducted through commercial simulation software (COMSOL Multiphysics 5.6). The experimentally measured values of elastic modulus, thickness, and width for the TMCLCEs were incorporated into the simulation, with a Poisson’s ratio of 0.49 applied in the calculations.

### Fabrication of TMCLCE pixels for multi-level encryption

Initially, TMCLCEs are affixed to a VHB adhesive film, offering a stable base. The temporary OHP films are then gently peeled away, leaving the CLCEs firmly adhered to the VHB layer. A transparent VHB layer is subsequently applied over the CLCEs, creating a flexible yet durable sandwich-like structure (refer to Fig. S6). This arrangement is crucial for enabling wavelength separation through mechanical stretching, which supports the photonic encryption functionality.

## Supplementary information

Supplementary Information

Supplementary Video S1

Supplementary Video S2

## Data Availability

The data that support the findings of this study are available from the corresponding author upon reasonable request.
