# Supplementary material for: Programmable optical encryption using thickness-controlled stretchable chiral liquid crystal elastomers
Source: Light Sci Appl. 2025 Mar 26;14:136. doi: 10.1038/s41377-025-01815-z (PMC11947189; doi:10.1038/s41377-025-01815-z)
Supplement: Supplementary file 1 — Supplementary Information [file 41377_2025_1815_MOESM1_ESM.pdf]

Supplementary Information

Programmable Optical Encryption Using Thickness-Controlled Stretchable Chiral Liquid Crystal Elastomers

Supplementary Information for

Programmable Optical Encryption Using Thickness-Controlled Stretchable Chiral Liquid Crystal Elastomers

*Seungmin Nam, Seohyun Woo, Ji Yoon Park and Su Seok Choi**

Department of Electrical Engineering, Pohang University of Science and Technology (POSTECH), Pohang, 37673, Korea

*Corresponding author: choiss@postech.ac.kr (Prof. Su Seok Choi)

Supplementary Note 1

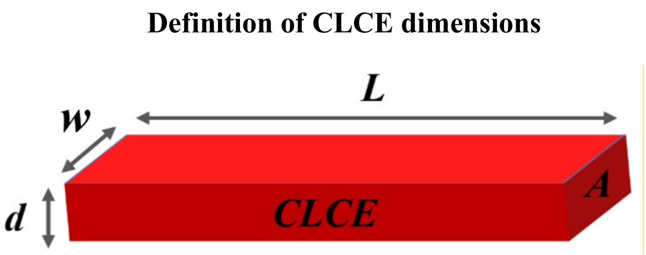

Mathematical derivation of CLCE thickness design

To begin, the elastic modulus of CLCE can be described as:

$$E = \frac{\sigma_t}{\varepsilon_t} = \frac{F/A}{\Delta L/L_0} = \frac{F/A}{(L_0 - L_1)/L_0} = \frac{F/(d \cdot w)}{(L_0 - L_1)/L_0} \quad (1)$$

where E is the elastic modulus of CLCE, σ_t is the tensile stress, ε_t is the tensile strain, F is the exerted tensile force, A is the cross-section area, ΔL is the length change, L_0 is the initial length, and L_1 is the length after stretching, d is the thickness, w is length that is orthogonal to the L and d.

Meanwhile, the central wavelength of the CLCE structural color can be represented as:

$$\lambda_c = n_{avg} \cdot p \quad (2)$$

$$\Delta\lambda_c = n_{avg} \cdot \Delta p = n_{avg} \cdot (p_0 - p_1) \quad (3)$$

where λ_c represents the central wavelength, $\Delta\lambda_c$ denotes the wavelength shift, n_{avg} is the average refractive index, p refers to helical pitch length of the CLCE, Δp indicates the helical pitch length change of the CLCE, p_0 is the initial pitch length, and p_1 is the pitch length after stretching.

Since the helical axis of CLCE is aligned along to the thickness direction (d-direction) of, the pitch length p is directly proportional to the thickness of the CLCE sample. Therefore,

$$p_1 = p_0 \cdot \frac{d_1}{d_0} \quad (4)$$

where d_0 represents the initial CLCE thickness and d_1 is thickness of the sample after stretching.

When a lateral tensile strain is applied perpendicular to CLCE helix, the strain in the thickness direction is related to Poisson's ratio as follows:

$$\nu = -\frac{\varepsilon_d}{\varepsilon_t} \tag{5}$$

$$\varepsilon_d = \frac{d_1 - d_0}{d_0} \tag{6}$$

where ν indicates the Poisson's ration of CLCE material and ε_d is the strain in the thickness direction.

Substituting equations (1), (4), (5), and (6) into equation (3),

$$\begin{aligned} \Delta\lambda_c &= n_{avg} \cdot \Delta p \\ &= n_{avg} \cdot (p_0 \cdot \nu \cdot \varepsilon_t) \\ &= \frac{n_{avg} \cdot p_0 \cdot \nu \cdot F}{E \cdot d_0 \cdot l} \end{aligned} \tag{7}$$

Consider the situation where the same amount of tensile force F is applied to the thickness modulated CLCEs in equation (7). Because the average refractive index n_{avg} is a material characteristic of the used CLC, it can be assumed that there is no significant difference. The initial pitch length p_0 can be assumed to have the same value because the initial central wavelength is set to have identical wavelength in all samples.

Assuming that Poisson's ratio ν also has the same value since there is no difference between samples as a result,

$$\Delta\lambda_c \propto \frac{1}{d} \tag{8}$$

Therefore, the effective color wavelength shifting amount relation should be similar as of the Equation (9).

$$\Delta\lambda_{c\_1}:\Delta\lambda_{c\_2}:\Delta\lambda_{c\_3} = \frac{1}{d_1}:\frac{1}{d_2}:\frac{1}{d_3} \quad (9)$$

where d_1, d_2, and d_3 are the thickness values of CLCEs, and $\Delta\lambda_{c\_1}$, $\Delta\lambda_{c\_2}$, and $\Delta\lambda_{c\_3}$ are the centra wavelength shifts of each CLCE sample.

In greater detail, the goal was to design color separation from same initial NIR (~800 nm) into Red (~650 nm), Green (~550 nm), and Blue (~450 nm), all under the same applied force. With the initial central wavelength of the CLCE set at approximately 685 nm, RGB color separation was possible when $\Delta\lambda_c$ of 150 nm, 250 nm, and 350 nm occurs in thickness varying samples $CLCE_1(d_1)$, $CLCE_2(d_2)$, $CLCE3(d_3)$, respectively.

Importantly, as derived from Equation (8), for the CLCE to effectively achieve wavelength separation, the following relationship should ideally be satisfied.

$$d_1:d_2:d_3 = 35:21:15 \quad (11)$$

Supplementary Note 2

Mathematical derivation of wavelength separation of TMCLCE under biaxial strain

Assuming equal stretching amounts are applied along the x-axis and y-axis to the CLCE, equip-biaxial strain and equip-biaxial stress can be defined as follows:

$$\varepsilon_b = \varepsilon_w = \varepsilon_L \quad (1)$$

$$\sigma_b = \sigma_w = \sigma_L \quad (2)$$

where ε_b represents the equip-biaxial strain, ε_x is uniaxial strain in the x-direction and ε_L is uniaxial strain in the L-direction. And σ_b represents the equip-biaxial tensile stress, σ_x is tensile stress in the x-direction and σ_L is tensile stress in the L-direction.

Furthermore, the CLCE can be assumed to be an incompressible material, and based on volume conservation, it can be expressed as follows:

$$(1+\varepsilon_w)(1+\varepsilon_L)(1+\varepsilon_d) = 1 \quad (3)$$

Also, the elastic modulus of CLCE can be described as:

$$E = \frac{\sigma_b}{\varepsilon_b} = \frac{F_L/A_L}{\Delta L/L_0} = \frac{F_L/A_L}{(L_0-L_1)/L_0} = \frac{F/(d \cdot w)}{(L_0-L_1)/L_0} \quad (4)$$

where E is the elastic modulus of CLCE, σ_L is the tensile stress, ε_d is the equip-biaxial tensile strain, F is the exerted tensile force, A is the cross-section area, ΔL is the length change, L_0 is the initial length, and L_1 is the length after stretching, d is the thickness, x is length that is orthogonal to the L and d.

Meanwhile, the central wavelength of the CLCE structural color can be represented as:

$$\lambda_c = n_{avg} \cdot p \quad (5)$$

$$\Delta\lambda_c = n_{avg} \cdot \Delta p = n_{avg} \cdot (p_0 - p_1) \quad (6)$$

where λ_c represents the central wavelength, $\Delta\lambda_c$ denotes the wavelength shift, n_{avg} is the average refractive index, p refers to helical pitch length of the CLCE, Δp indicates the helical pitch length change of the CLCE, p_0 is the initial pitch length, and p_1 is the pitch length after stretching.

Since the helical axis of CLCE is aligned along to the thickness direction (d-direction) of, the pitch length p is directly proportional to the thickness of the CLCE sample. Therefore,

$$p_1 = p_0 \cdot \frac{d_1}{d_0} \quad (7)$$

where d_0 represents the initial CLCE thickness and d_1 is thickness of the sample after biaxial stretching.

When biaxial tensile strain is applied perpendicular to CLCE helix, the strain in the thickness direction can be expressed as follows:

$$\varepsilon_d = \frac{d_1 - d_0}{d_0} \quad (8)$$

Assuming an isotropic polymer with volume conservation, when an equip-biaxial tensile strain is applied perpendicular to the CLCE helix, the thickness after biaxial stretching can be expressed as follows:

$$d_1 = \frac{1}{(1+\varepsilon_b)^2} \cdot d_0 \quad (9)$$

Therefore, the pitch length after biaxial stretching can be expressed as follows based on equations (4) and (6):

$$p_1 = p_0 \cdot (1 + \varepsilon_b)^2 \quad (10)$$

Substituting equations (1), (4), (5), and (6) into equation (3),

$$\Delta\lambda_c = n_{avg} \cdot \Delta p$$

$$= n_{avg} \cdot p_0 \cdot \frac{F \cdot (2 \cdot E \cdot d_0 \cdot l + F)}{E \cdot d_0 \cdot l \cdot (E \cdot d_0 \cdot l + F)^2} \quad (11)$$

Assuming all material properties except for thickness are identical and the same force is applied, when plotting the given equation with the x-axis representing the initial thickness of the CLCE (d_0) and the y-axis representing the wavelength shift ($\Delta\lambda_c$) the following relationship is obtained:

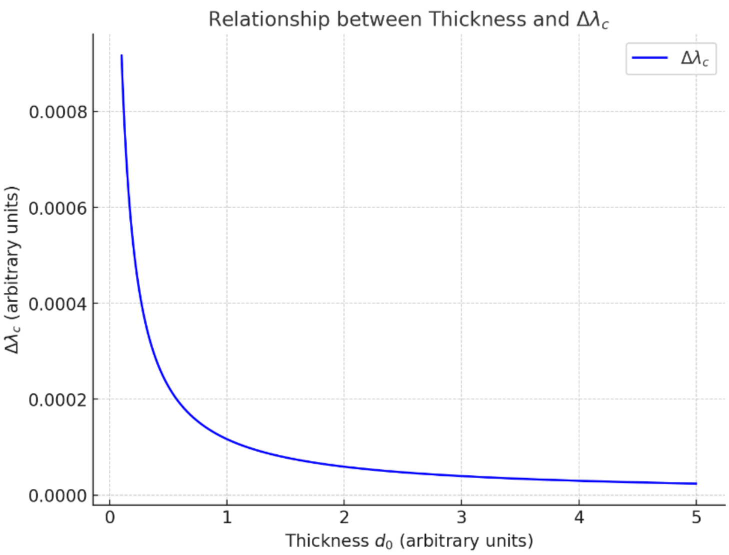

Figure S1. The variation trend of wavelength shift with respect to the thickness of the CLCE.

Therefore, when biaxial stretching is applied to the CLCE, the thickness and wavelength shift exhibit an inversely proportional relationship. This confirms that wavelength separation can be achieved by utilizing thickness modulation.

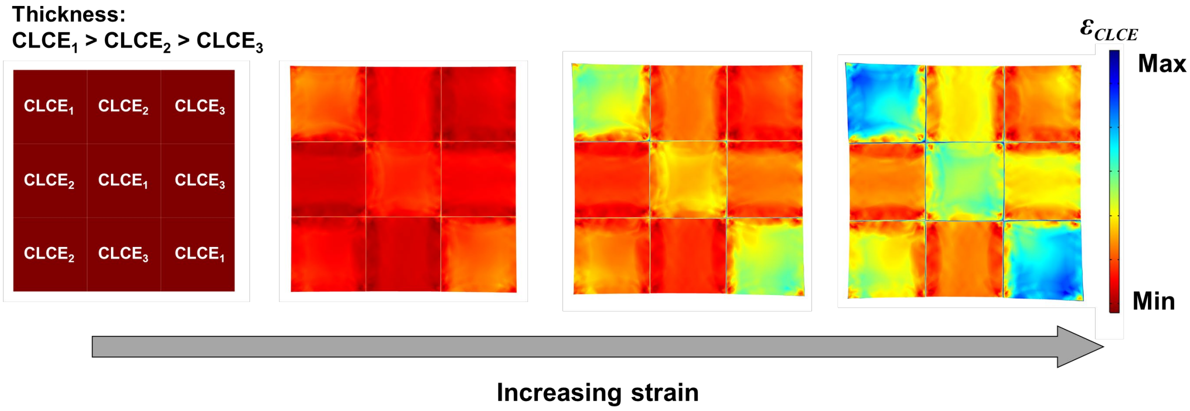

Figure S2. FEA simulation results of strain distribution in thickness-modulated CLCEs with 3 by 3 pixel configuration under biaxial strain.

Table S1. Amounts of materials used for preparing the thickness modulated chiral liquid crystal elastomers (TMCLCEs).

Unit: wt%

Initial wavelength	RM257	LC756	EDDET	PETMP	Irgacure 651	DPA
Visible (Fig. 1~ 4)	73.83	3.57	17.72	4.14	0.50	0.23
IR (Fig. 5)	73.72	2.99	18.28	4.28	0.50	0.24

Table S2. Summary of recent research on photonic encryption devices using tunable structural color.

Triggering Method	Tuning Mechanism	Tuning Range	Discrete Pixel Structure	Multi-wavelength separation	Ref
Mechanical deformation and moisture	Wrinkling induced by deformation mismatch, erased by moisture	Visible (red to blue)	X	X	[1]
Thermal stimulation and mechanical deformation	Dynamic exchange reactions between disulfide bonds	400 - 700 nm	X	X	[2]
Thermal stimulation	Thermochromic adjustments via crosslinked network	450 - 650 nm	X	O	[3]
Thermal stimulation	Helical pitch changes via thermo-responsive CLCs	500 - 600 nm	O	X	[4]
Mechanical deformation and chemical stimulus	Structural periodicity and pigmentary change	450 - 700 nm	X	X	[5]
Mechanical deformation, light exposure and thermal stimulation	Helical pitch changes and dynamic bond exchange	Visible (red to blue	X	X	[6]
Mechanical deformation	Helical pitch changes via mehchanical deformation	IR to Visible	X	X	[7]
Mechanical deformation	Helical pitch changes via mehchanical deformation	IR to Visible	O	O	This study

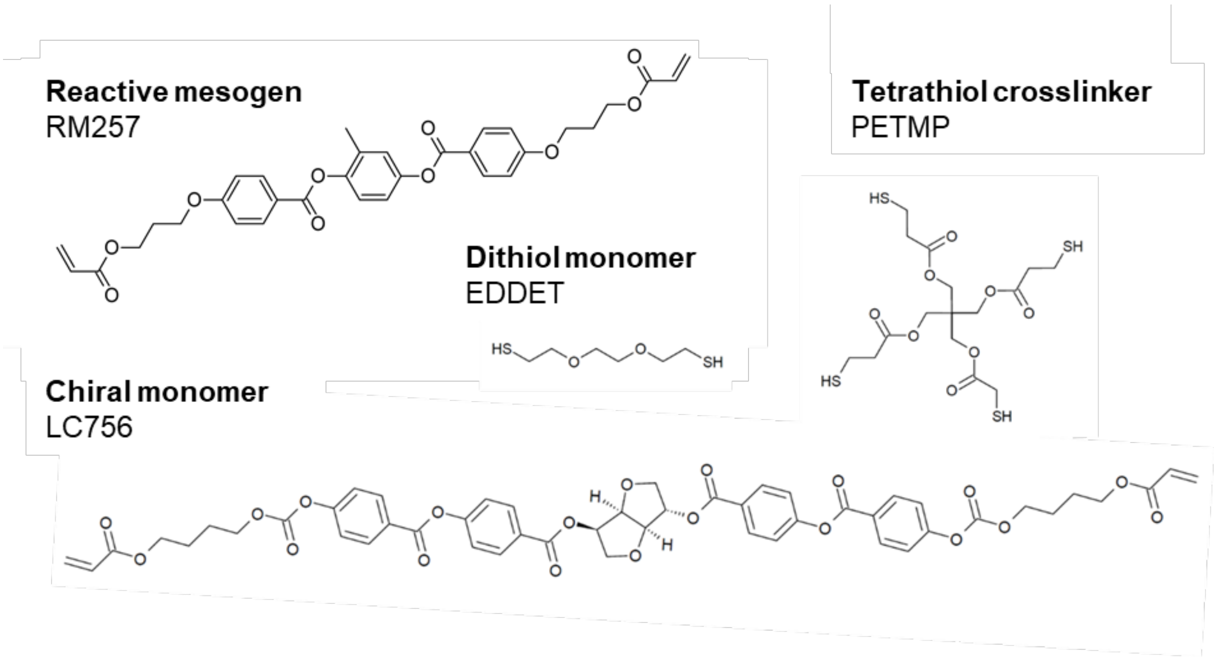

Figure S3. Molecular structure of materials used to fabricate thickness modulated CLCEs.

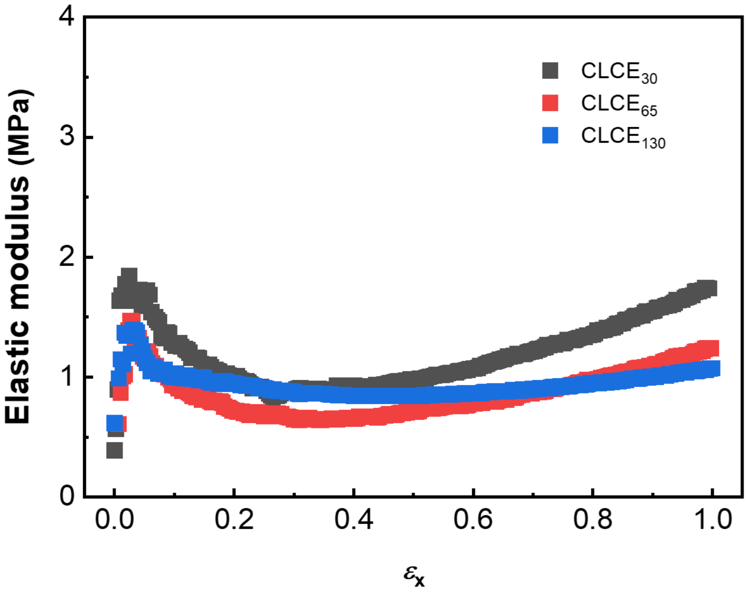

Figure S4. Elastic modulus variation of thickness-modulated CLCE with respect to uniaxial tensile strain.

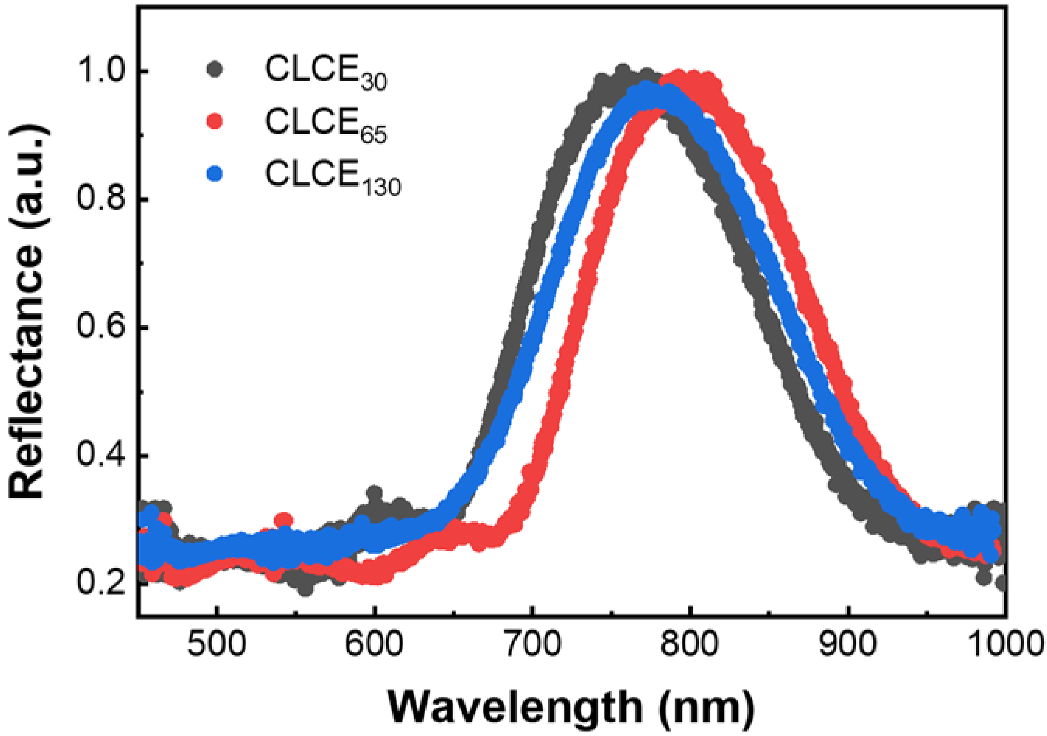

Figure S5. Reflection spectra of CLCEs with central wavelengths located in the IR region, as used in the pixel-configured encryption device shown in Figure 5.

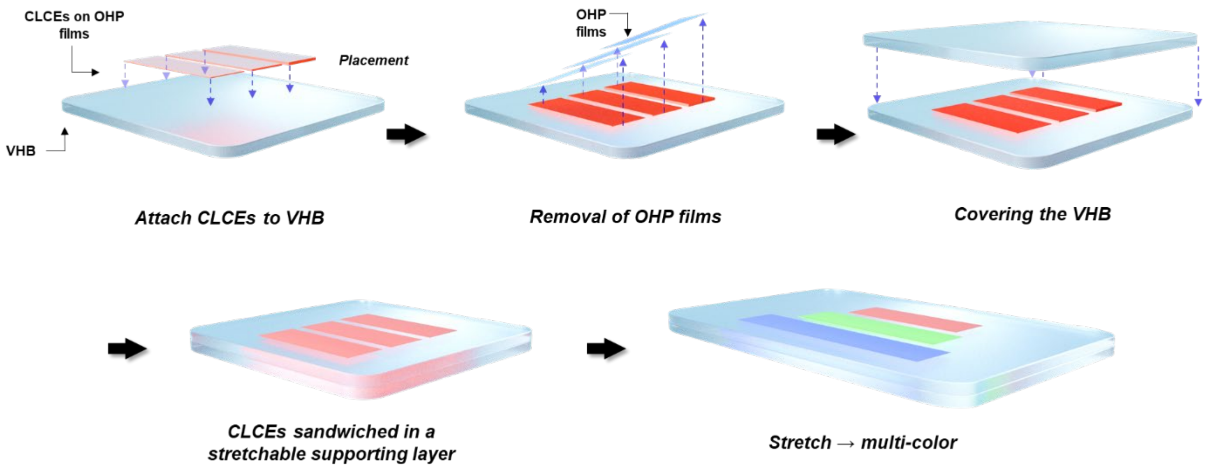

Figure S6. Fabrication process of stretchable multi-level photonic encryption device using thickness modulated CLCEs and stretchable supporting layer.

a **Base64 index table**

Val	Char	Val	Char	Val	Char	Val	Char
0	A	16	Q	32	g	48	w
1	B	17	R	33	h	49	x
2	C	18	S	34	i	50	y
3	D	19	T	35	j	51	z
4	E	20	U	36	k	52	0
5	F	21	V	37	l	53	1
6	G	22	W	38	m	54	2
7	H	23	X	39	n	55	3
8	I	24	Y	40	o	56	4
9	J	25	Z	41	p	57	5
10	K	26	a	42	q	58	6
11	L	27	b	43	r	59	7
12	M	28	c	44	s	60	8
13	N	29	d	45	t	61	9
14	O	30	e	46	u	62	+
15	P	31	f	47	v	63	/

b **CSS decryption table**

Row 1		Row 2		Row 3		Row 4	
Input	Output	Input	Output	Input	Output	Input	Output
A	E	A	Q	A	X	A	Z
B	F	B	L	B	C	B	E
C	L	C	S	C	I	C	L
D	C	D	G	D	J	D	H
E	W	E	F	E	L	E	S
F	G	F	E	F	M	F	D
G	A	G	W	G	Q	G	A
H	D	H	Y	H	T	H	U

Figure S7. Decryption table for stretchable multi-level photonic encryption in Figure 5. (a) Base64 index table for converting the binary code into characters. (first step decryption). (b) Lab-made CSS decryption table created in the lab for additional security enhancement. (second step decryption).

Supplementary Video S1 | Thickness modulated CLCEs under uniaxial stretching (FEA Simulation)

Supplementary Video S2 | Stretchable multi-level photonic encryption system using TMCLCE pixels

References

[1] Lin, R. et al. Biomimetic photonic elastomer exhibiting stress/moisture reconfigurable wrinkle-lattice for reversible deformation information storage. *ACS Nano* 18, 13346–13360 (2024).

[2] Hussain, S. & Park, S. Photonic cholesteric liquid-crystal elastomers with reprogrammable helical pitch and handedness. *ACS Appl. Mater. Interfaces* **13**, 59275–59287 (2021).

[3] Wen, X. et al. Bio-inspired cholesteric phase cellulose composite with thermochromic and circularly polarized structural color for multilevel encryption. *Adv. Funct. Mater.* **2308973**, 1–10 (2023).

[4] Zhan, X. et al. Wavelength-tunable circularly polarized laser arrays for multidimensional information encryption. *Adv. Opt. Mater.* **11**, 1–8 (2023).

[5] Zhang, S. et al. Reversible information storage based on rhodamine derivative in mechanochromic cholesteric liquid crystalline elastomer. *Adv. Funct. Mater.* **33**, 1-10 (2023).

[6] Liu, J. et al. Visible-light-programmed patterning in dynamically bonded cholesteric liquid crystal elastomer. Nat. Commun. 15, 1–11 (2024).

[7] Zhang, X. et al. Mechanically Tunable Circularly Polarized Luminescence of Liquid Crystal-Templated Chiral Perovskite Quantum Dots. Angew. Chemie Int. Ed. 63, (2024)
